# Exploring diversity: a review of animal models for investigating variations in sex characteristics

**DOI:** 10.3389/fped.2025.1430983

**Published:** 2025-11-21

**Authors:** Gabriela de Faria Oliveira, Thomas Niepsuj, Arushi Gupta, Anthony P. Auger

**Affiliations:** 1 Wisconsin National Primate Research Center, Madison, WI, United States; 2 WNPRC and Department of Psychology, University of Wisconsin-Madison, Madison, WI, United States

**Keywords:** sex differences, animal models, sex characteristics, sexual differentiation, VSCs

## Abstract

This review examines mechanisms across various organisms to enhance comprehension of Variations in Sex Characteristics (VSCs) and suggests suitable models for investigation. VSCs are the result of a collection of congenital conditions characterized by atypical development of both internal and external reproductive structures. These conditions may arise from variations in genes, developmental processes, and hormonal influences. Animal models are crucial for studying VSCs due to their ability to provide valuable insights into the underlying mechanisms in which sexual differentiation exists. Comparative research across species has further emphasized the nature of certain sex-determining genes and pathways, providing a broader understanding of the evolutionary conservation of sexual differentiation mechanisms. By integrating findings from diverse animal models, encompassing the genetic, molecular, and comparative perspective, this review seeks to provide a cohesive understanding of the complex processes underlying VSCs in animals, with implications for translational research and potential treatments.

## Introduction

Phenotypic sex refers to the observable characteristics of an organism, influenced by its genetic makeup, which directs the development of internal ducts, external genitalia, and reproductive organ systems. These traits further shape the organism's reproductive maturity and behavior through subsequent hormonal and developmental signaling processes—with significant variation across species (1). Variability is fundamental in the processes of evolution; thus, choosing a model organism that follows a relevant mechanism is important in better understanding sexual differentiation. Here, we will examine organisms, to not only better understand possible mechanisms at work, but also suggest and compile animal models that may be helpful in better understanding some Variations of Sex Characteristics (VSCs) and their potential impact on brain and behavior.

Sexual differentiation, in mammals, is typically the result of the sex chromosome complement of the organism (XX or XY), and multiple molecular events that direct the development of the gonad primordium into a testis (due to the consequences of the presences of Sry and related genes mentioned below in the Y chromosome) or an ovary (when Sry is not present and XX chromosomal signaling directs organized development) (2). The sex chromosome sets the stage for the development of any typical male- or female-specific physical or behavioral characteristic in response to hormones produced by the gonads, also known as sexual differentiation (3). This intricate process involves a series of events that begin with genes specific to sexual differentiation, followed by hormonal cascades that direct the differentiation of reproductive organs, gametes, secondary sex characteristics, and ultimately, the brain (4). This complex process is not always standard, as conditions may arise from variations in genes, developmental processes, and hormonal influences (5), and may result in atypical gonads, or atypical phenotypic sex (6). Some people affected by VSCs may exhibit external genital ambiguity at birth, while others may manifest postnatal virilization, delayed puberty, and/or infertility later in life (7).

Over time, a variety of phrases have been used to describe the uncommon development of phenotypic sex. In the past, to recognize differences in sex development, some groups adopted “Disorders of Sex Development” or DSD (6). Current terminology includes “intersex”, “differences of sex development”, “variations in sex characteristics”, “divergence of sex development”, “intersex variations” and “diverse sex development” (8). To note, some consider the use of medical language like “DSD” or “conditions” as pathologizing, as it implies a need for potentially unnecessary medical interventions, and instead may prefer the use of the term “intersex” (9). Therefore, it is important to recognize that some terms are stigmatizing, and to consider the terms used by the individual or group. For this review, we will use the terminology VSCs, but that is not an attempt to standardize or suggest it as a singular umbrella term. VSCs communities face stigma, and it is important to recognize the wants and needs of each community, over generalizing or assuming what their wants and needs may be. Different people with VSCs may use different terms, and researchers and physicians must respect these decisions. The goal of this work is to consolidate information in a way that better advocates ways of studying VSCs, to provide better consensual outcomes for those with a VSCs.

Animal models are crucial for studying VSCs because they provide valuable insights into the underlying mechanisms by which sexual differentiation occurs. Comparative research across species has further emphasized sex-determining genes and pathways, providing a broader understanding of the evolutionary conservation of sexual differentiation mechanisms. Moreover, animal models have offered valuable insights into the neurobiological basis of sexual differentiation, being pivotal to the investigation of the neural control of sex differences in behaviors, synaptic connectivity, and brain development. In this review, we will highlight current understandings of sexual differentiation in animal models, by focusing on the molecular, hormonal, and comparative perspectives. By integrating findings from diverse animal models, this review seeks to provide a supportive understanding of the complex processes underlying sexual differentiation and VSCs in animals, with implications for translational research and potential treatments.

## Sex chromosomal and genetic VSCs

In organisms characterized by XX and XY sex chromosomes, a key objective is to discern how each of these sex chromosomes influences phenotypic traits (10). One of the imbalances caused by X and Y genes pertains to the sexual differentiation of the gonads. Early during development, the bipotential gonad has the capacity to be feminized or masculinized depending upon the activity of particular genes. Classically, gonadal differentiation begins to unfold during fetal development when the sex-determining region of the Y chromosome (Sry) is activated (11, 12). Sry is homologously conserved in humans (12) and is an embryonically active gene responsible for the differentiation of the testes and the development of masculinized characteristics (13). The landmark discovery of the Sry gene in a mouse model opened the doors to discovery of several other regulatory genes that are important in sexual differentiation. It was later determined that there are ovary related genes, such as R-spondin1 (RSPO1), WNT family member 4 (WNT4), catenin beta 1 (Ctnnb1) and forkhead transcription factor L2 (FOXL2) and testis related genes (SRY, SOX9 and FGF9) required for differential gonadal development (14). While these particular genes are important, we will also highlight others that have contributed to VSCs below. Additionally, some of these genes are regulated sequentially to induce gonadal differentiation. Furthermore, studies have shown that Sry is upregulated briefly, thereby increasing Sox9, which then increases Fgf9 (15), which feedback on Sox9, resulting in masculinization of the gonad. Alterations in the levels or function of these genes can contribute to VSCs. For example, SOX9 and SOX3 are essential in gonad development, and mice with Copy Number Variations (CNVs) of these genes (16) can lead to a rare form of gonad tissue that contains both ovarian and testicular elements, similar to ovotesticular disorder in humans. Another gene involved in the ovotesticular disorder is the NR2F2, and previous research has shown that the NR2F2 gene acts as a “pro-ovary” factor in the primordial gonad, promoting ovary identity by repressing genes involved in testis determination (17). This mechanism can be studied further in knockout and knock-in mice with NR2F2 mutations (18). Like NR2F2, RSPO1 is crucial to ovarian development, and RSPO1 knockout mice may exhibit altered gonadal development, including ovotestes or other relevant phenotypes (19). These findings appear to recapitulate the functional role of these genes on human gonadal differentiation. Specifically, RSPO1 is expressed at higher levels in developing human ovarian tissue compared to testis, and mutations in RSPO1 results in ovotesticular development (20). These data are consistent in that RSPO1 is involved in ovarian development in humans and rats. NR2F2 is more complicated to discern its impact on tissue development as it is considered a steroid receptor function regulatory protein. Therefore, disrupting the functional role of NR2F2 is likely to impact hormone sensitive differentiation and function of tissues and cellular pathways. It is important to recognize that many genes involved in VSC diagnoses also regulate the differentiation of other tissues and signaling pathways. That is, most of these genes are not tissue specific and can impact the function of other organs and processes. For example, NR2F2 is important for cardiac function (21), and RSPO1 influences WNT signaling and has been implicated in dermal disease and cancer (22). Therefore, not only do these animal models allow us to interrogate functional pathways involved in VCSs, but reveal how these genes impact other physiological systems, with the hope of better understanding how to ameliorate negative consequences in humans. These are all examples of changes in specific genes, but full chromosomal aneuploidy and nondisjunction genetic manipulations establishes additional models for VSCs that have the complete addition or lack thereof of specific X or Y chromosomes, such as Klinefelter syndrome (23), Turner syndrome (24) and 45,X/46,XY mosaicism (25).

Other molecular pathways, such as hedgehog (HH) morphogens, are involved in the development of male primary and secondary phenotypes. The Glioma-Associated Oncogene Homolog (GLI) proteins are key transcription factors in the Hedgehog signaling pathway. The modulation of GLI proteins in mouse models has been instrumental in elucidating the functions of GLI proteins and their implications in the development of the genitals and urinary tracts. Previous research established that a decrease in Hedgehog pathway activity due to reduced *Gli2*/*Gli3* levels results in significant malformations in the hindgut and urogenital sinus. *Gli2*/*Gli3* mouse models reveal critical information about the molecular factors involved in malformations and developmental processes, but there are challenges in developing pertinent animal lines because of the lethal nature of these abnormalities (26). However, more recent models with a compound mutant (*Gli2*^+/−^; *Gli3*^Δ699/+^) convey urogenital sinus malformations into adult life stages without posing life-threatening complications. As mutations in these genes also impact forebrain development, it is currently being investigated if *Gli2*^+/−^; *Gli3*^Δ699/+^ mutations have consequences for behavior. It is important to note that Gli2 and Gli3 protein in mice have around 95% similarity in amino acid structure, and more protein alignment with humans have around 86% similarity in structure making it a good model to recapitulate human Gli2/Gli3 dysfunction in rodents. As Gli2/Gli3 modifies brain and neuronal development, these models give insight into how they may alter mental health risk within the VSC community. Therefore, these models can be employed for behavioral and physiological assays to connect the multifaceted peripheral, central, and cognitive changes those with VSCs may face.

### Connections to clinical practice

Collectively, these genetic and chromosomal models have been pivotal in bridging basic science discoveries with clinical applications. The identification of key genes such as *SRY*, *SOX9*, *RSPO1*, and *NR2F2* in mouse and human studies has directly informed the molecular diagnosis of VSCs, improving both the accuracy and the timing of clinical interventions. For example, murine models with *SOX9* or *RSPO1* disruptions have elucidated the molecular cascade that underlies gonadal dysgenesis, guiding the development of targeted genetic screening panels now used in pediatric endocrinology and reproductive medicine. Likewise, comparative analyses of aneuploidy models, such as *XXY* (Klinefelter) and *XO* (Turner) mice, have provided critical insights into chromosomal dosage effects on growth, fertility, and neurocognitive function; knowledge that informs long-term care strategies for individuals with sex chromosome aneuploidies.

Beyond genetics, the study of signaling pathways such as Hedgehog (*GLI2/GLI3*) in animal models has expanded our understanding of urogenital malformations and their neurological correlates. These findings have clinical implications for the multidisciplinary management of patients with concurrent reproductive and neurodevelopmental alterations. Furthermore, such models underscore that many genes implicated in VSCs are pleiotropic, influencing multiple organ systems, including cardiac and dermal tissues. Ultimately, the use of genetically engineered animal models enables researchers to dissect the causal links between molecular perturbations and phenotypic outcomes in ways that are not possible in human studies. These insights refine clinical diagnostics and counseling, inform personalized hormone management strategies, and contribute to ethical frameworks that prioritize evidence-based and patient-centered decision-making in VSC care.

### Limitations and future directions

Although chromosomal and gene-targeted animal models have greatly advanced understanding of gonadal differentiation, several limitations hinder direct translation to clinical settings. In murine systems, for example, the timing of *Sry* activation and the cascade of *Sox9* and *Fgf9* expression occur over a much shorter developmental window than in humans, which may distort the relative contributions of genetic dosage and timing to gonadal differentiation. Moreover, genes implicated in VSCs often exert pleiotropic effects beyond the gonads; thus, global knockout models can conflate primary gonadal mechanisms with systemic phenotypes. Hedgehog-pathway mutants (*Gli2*, *Gli3*) illustrate another constraint, as severe morphogen disruption frequently results in embryonic lethality or profound malformations that preclude behavioral or neuroendocrine assessment in adulthood. Similarly, while *XXY* and *XO* mouse lines replicate certain aspects of Klinefelter and Turner syndromes, they incompletely reproduce the neurocognitive, stature, and metabolic profiles observed in humans.

Importantly, genetic manipulations cannot capture the lived experiences associated with the minority stress model or the impact of human stigmatization, which are integral to understanding psychosocial outcomes in individuals with VSCs. Nevertheless, they remain invaluable for elucidating the molecular and hormonal signaling pathways that underlie these conditions. Such mechanistic insights can ultimately inform a more compassionate and evidence-based clinical approach, fostering collaboration among endocrinologists, geneticists, and behavioral health specialists. To bridge the translational gap, future research should adopt conditional and stage-specific alleles, such as Cre/lox or CRISPR interference of *RSPO1*, *FOXL2*, and *SOX9* within Sertoli or granulosa lineages, to disentangle gonadal from systemic effects. Developing hypomorphic *Gli2/Gli3* lines that preserve viability while sustaining urogenital phenotypes into adulthood would further enhance translational value. Additionally, nonhuman primate models offer an opportunity to explore how hormonal and neurobiological factors interact within a social and developmental context that more closely parallels human physiology and behavior, providing a crucial link between molecular findings and real-world clinical applications.

## Steroid biosynthesis and VSCs

Although chromosomal variations (and modulation) can play a direct role in the manifestation of VSCs, they can also influence VSCs via altered cellular development impacting the regulation of the steroid synthesis pathways. As stated above, genes influence the development of the gonad, and if this development is atypical, the cell types within the gonad may have atypical steroid production and/or responses. Research has clearly indicated that alterations in sex chromosomes can result in VCSs directly or through altered tissue development that further disrupts the cells producing steroid hormones. These hormones have a vast array of signaling pathways (27), and play a fundamental role in sexual differentiation and development (28, 29). Thus, when these pathways are modulated, genetically or systematically, there can be changes in masculinization or feminization across tissue types at different developmental stages (30). Whether direct disruption of the steroid synthesis or downstream impacts of the disrupted signaling, a variety of mechanisms can be the causal factor of VSCs. These changes in synthesis and signaling can happen via a multitude of mechanisms. Thus, recreation of each mechanism in a model organism would be a powerful way to recapitulate physiology and investigate this physiology in a translational manner.

The androgen receptor null mouse is one such example, as the elimination of a steroid response element mirrors what we see in some instances of Androgen Insensitivity Syndrome (AIS), thus is an excellent model to understand VSCs and androgen receptor impacts on other systems (31). This model can not only give insights into VSC experiences, but from a developmental perspective, parse apart the influence of X and Y chromosome gene expression that directs or is independent of classical androgen signaling. Using androgen receptor, or other steroid receptor, knockout models (32) has provided information on the role of these receptors in a variety of systems across physiology and behavior, including social and cognitive development. Such findings are especially relevant to the AIS community, as they highlight the widespread and nuanced impact of disrupted androgen signaling across the lifespan. However, due to the complex etiology of AIS, current knockout models may not fully capture the nuance of said etiology.

**Figure 1:**
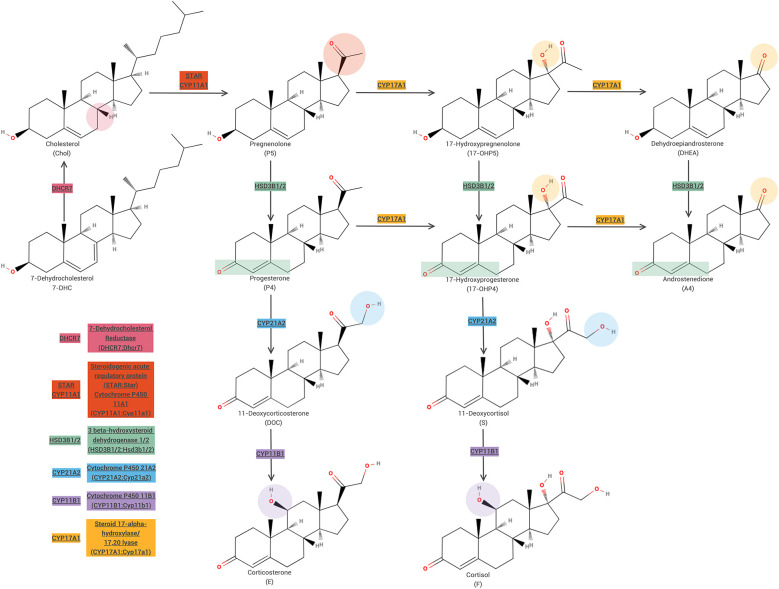
Steroid synthesis pathway highlight regulatory enzymes common in CAH.

Due to the complexity of steroid signaling, alterations at various points along the pathways can produce similar VSCs, yet result in different physiological effects. Specifically, modifying the pathway at an earlier stage (upstream) is likely to induce pronounced changes in both VSCs manifestations and physiological outcomes compared to modifications at a later stage (downstream). For example, mutations in Star, an important factor that guides cholesterol into the mitochondria to initiate the steroid synthesis pathway, can result in Congenital Adrenal Hyperplasia (CAH) (33), but modulations to Cyp11a1, the next protein to facilitate the synthesis of steroid hormones, can also result in CAH (34), but to a different degree (Figure 1). Further down the steroid synthesis pathway, other modulations can cause CAH such as Mamld1 knockdown (35) or modulations to HSD3B2 (36). This example in which modulation of steroid synthesis at various stages being the causal factor for VSCs is not limited to CAH.

The choice of model organism in research often depends on the specific aspect of a disorder or mechanism one aims to investigate. In the context of hormonal manipulations, researchers typically expect significant impacts on physiological or behavioral phenotypes, depending upon the time of hormonal alterations. However, the extent and nature of these changes can also vary greatly depending on the level at which steroidogenesis is disrupted. For instance, although both STAR and HSD3B2 mutations can lead to congenital adrenal hyperplasia (CAH), they result in markedly different profiles of hormone production and signaling. In contrast, targeting downstream enzymes like HSD3B2 allows for more selective disruption, potentially preserving peripheral functions while still modeling specific aspects of hormonal regulation. Thus, the selection between these models should be guided by the degree of systemic vs. localized impact desired in relation to the research question.

The timing of hormonal changes, and their experimental modulation, is a critical factor when modeling VSCs. One foundational concept in this context is the masculinization programming window: a well-defined, temporally restricted period during fetal development in which androgen activity is essential for the proper differentiation of both reproductive structures and sexually dimorphic brain circuits (37). Disruptions to androgen signaling during this window can result in permanent alterations in genital morphology, secondary sexual characteristics, and brain organization. This window has been robustly characterized in rodent and nonhuman primate models (38), and is highly conserved across species. For example, studies by Welsh et al. (30), Scott et al. (39), and Welsh et al. (37) demonstrated that androgen exposure limited to this window dictates key developmental outcomes such as penile length and urethral positioning, features directly relevant to conditions like hypospadias.

All things considered, understanding sex differences in brain structure and behavior requires careful consideration of both the hormonal milieu and the timing of exposure. Rodent models, with their well-characterized perinatal testosterone surge, are particularly useful for parsing the organizational effects of androgens. However, brain sexual differentiation in rodents occurs later than in humans and nonhuman primates, primarily during late gestation and early postnatal life. This temporal mismatch underscores the importance of model selection, especially when investigating neurodevelopmental aspects of VSCs that originate during the earlier masculinization window seen in primates and humans.

### Connections to clinical practice

Findings from steroidogenic enzyme and receptor knockout models have been instrumental in refining our understanding of hormonal disorders that give rise to VSCs and have provided a foundation for translating basic endocrinology into clinical care. For example, studies using *Star*, *Cyp11a1*, and *Hsd3b2* mutant mice have delineated critical steps in steroidogenesis that mirror the enzymatic defects seen in human congenital adrenal hyperplasia (CAH). These models have directly informed the timing, dosage, and composition of glucocorticoid replacement therapies in pediatric endocrinology, where early intervention is essential to prevent adrenal crises and optimize growth and pubertal outcomes. Similarly, *Mamld1* and androgen receptor null models have advanced our understanding of androgen insensitivity and under virilization syndromes, offering mechanistic insights that inform individualized hormone therapy and fertility preservation strategies.

Beyond hormonal replacement, animal models of disrupted steroid biosynthesis have also been valuable for predicting systemic effects of altered steroid metabolism. For instance, studies in rodent models have revealed that perturbations in the androgen–estrogen balance during critical developmental windows can permanently reorganize neural and metabolic circuits. This has translational relevance for individuals with VSCs who may present with neurobehavioral or metabolic comorbidities later in life. Understanding these systemic consequences helps clinicians anticipate and monitor secondary effects of hormone therapy, such as mood alterations, metabolic shifts, or cardiovascular risks, thereby promoting more comprehensive long-term care.

Importantly, animal research has emphasized that the timing of steroid exposure is as critical as the dosage. The concept of a “masculinization programming window,” has reshaped clinical perspectives on when and how hormonal therapies should be administered during development. For example, insights from these models have informed guidelines for puberty modulation or hormonal replacement in adolescents with VSCs, ensuring interventions align more closely with sensitive neuroendocrine periods that support healthy cognitive and emotional outcomes.

Overall, research on steroid biosynthesis in animal models continues to serve as a bridge between molecular endocrinology and patient care. These models help refine diagnostic biomarkers, identify new therapeutic targets, and inform evidence-based clinical decisions. By integrating developmental, endocrine, and behavioral perspectives, translational work derived from these systems is contributing to a more nuanced, compassionate, and physiologically grounded approach to managing VSCs in clinical practice.

### Limitations and future directions

Despite their translational importance, steroidogenic models also face limitations. Early-pathway knockouts can result in perinatal lethality due to complete loss of steroidogenesis, preventing assessment of pubertal or behavioral outcomes (40). This is exemplified in rodent models of Smith–Lemli–Opitz syndrome, in which complete loss of *Dhcr7* (*Dhcr7⁻/⁻*) is lethal (40, 41), complicating translational modeling of this condition. However, it is important to note that the relationship between gene disruption and viability is not absolute**.** In humans, individuals with Smith–Lemli–Opitz syndrome survive despite severe reductions, but not complete absence, of DHCR7 enzyme activity, suggesting that modulated signaling, partial loss-of-function mutations, and isoform-specific effects can sustain minimal but functionally sufficient steroidogenesis. This nuance highlights that knockouts and hypomorphic or missense mutations represent fundamentally different biological contexts, with the latter often providing a more accurate reflection of the human condition. Indeed, hypomorphic mouse models (42) and conditional knockouts (43) have been instrumental in revealing age-related changes and mechanisms that can be therapeutically targeted. Moreover, studies using adeno-associated viral (AAV) vector–mediated rescue demonstrate partial phenotypic recovery (44), offering valuable translational insight into how restoring DHCR7 activity might ameliorate Smith–Lemli–Opitz syndrome in humans. Such findings emphasize the need for graded, isoform-specific, and temporally controlled models that better capture the spectrum of human steroidogenic disorders rather than binary knockout systems alone.

Rodents additionally differ from humans in adrenal zonation, fetal gonadal steroid output, and placental aromatase activity, complicating extrapolation of prenatal and perinatal androgen–estrogen milieus (45). To address some of these limitations, nonhuman primate models offer a uniquely translational platform for investigating steroid biosynthesis and signaling in VSCs (46). Compared to rodents, primates share a more homologous organization of the hypothalamic–pituitary–gonadal axis, a similar temporal pattern of adrenal and gonadal steroid production, and overlapping enzymatic pathways governing androgen and estrogen synthesis (47). Importantly, fetal and pubertal hormone surges in species such as the common marmoset (*Callithrix jacchus*) and rhesus macaque (*Macaca mulatta*) occur on developmental timelines comparable to those in humans, allowing direct assessment of how transient endocrine manipulations influence reproductive and neural outcomes (48, 49).

Future studies should leverage this translational potential by integrating NHP research with advanced genetic and pharmacological tools. For instance, controlled suppression or stimulation of steroidogenic enzymes during defined developmental windows could clarify how disruptions in androgen or estrogen synthesis affect organ differentiation, puberty onset, and cognitive–emotional function. Such designs would align directly with clinical questions about the optimal timing and safety of hormone therapies in individuals with VSCs.

## Sexual differentiation of the brain

Sexual differentiation is a fundamental process that leads to the development of distinct male and female phenotypic characteristics. Research has shown that sexual differentiation of the brain is largely driven by hormonal influences occurring perinatally in rodents and prenatally in primates (50). Hormones, such as testosterone and estradiol, play essential roles in controlling brain and sex differences in behavior (51). For instance, testosterone secreted by the testes during the perinatal period predominantly initiates the sexual differentiation of the male mammalian brain (52). Additionally, the pubertal period has shown to be a second period of organization for sex differences in behavioral expression in males (52, 53) as well as in females, in which estrogen plays a crucial role in the beginning of puberty as it is required for the full development of female-typical behavior (54).

Sex differences in the brain have been extensively researched, with animal models providing an understanding to the underlying mechanisms of sexual differentiation. Before the 1900s, the understanding of sexual differentiation was limited, with early observations focusing on anatomical differences between males and females. In the mid-1900s, Jost's experiments in rabbits established the dogma that genetic sex plays a crucial role in determining whether the gonad develops into a testis or an ovary, and once a testis is formed, its hormones influence on the male sex phenotype (55–57). During this time it was generally accepted that the brain in both sexes is very similar, if not identical, and that differences in behavior are only reflected at the neurophysiological level. However, studies on birds and rats (58, 59) contributed to the identification of small sex differences in specific aspects of neuronal morphology and a shift in thinking took place when sex differences in the volume of song control nuclei of birds such as canaries and zebra finches were reported (60). Following the identification of volumetric sex differences within the hypothalamus of rats, it was later shown that humans also have structural sex differences in similar brain regions (61), such as the third interstitial nucleus of the anterior hypothalamus (62).

After the 1900s, advancements in molecular biology revolutionized the study of sexual differentiation. Studies in marsupials and mice showed that anatomical differences between male and female embryos start before gonadal differentiation, thus indicating a hormone-independent pathway for sex dimorphisms (63). Another major contribution comes from studies on birds. Birds are valuable animal models for studying sex differences in the brain due to their unique characteristics and evolutionary history. Studies have shown that somatic sex identity in chickens is cell autonomous, indicating that sexual differentiation may occur independently of hormonal influences in this animal model (64). This finding challenges the traditional mammalian model of sexual differentiation, whereas embryos are considered to be sexually indifferent until the transient action of the Sry gene starts gonadal differentiation. Avian models have also been instrumental in studying endocrine and neurodevelopment. Specifically, the study of sex dimorphic gene expression in the brains of songbirds has provided valuable insights into sex differences in behavioral expression, learning, and neuroplasticity. While songbirds are not ideal models for studying urological or urogenital conditions directly, their unique contribution lies in elucidating the neural and molecular mechanisms underlying sex differences, making them an invaluable model for understanding the complex signaling networks involved in VSCs.

Delineating the genetic vs. the systemic and hormonal contributions to sexual differentiation can be challenging, so very specific genetic manipulations in animal models can allow investigators to focus on gene-directed mechanisms, independent of systemic influence (65–67). The Four Core Genotype (FCG) rodent model is a great example of such genetic manipulation and was created by merging the elimination of the Sry gene from the Y chromosome (resulting in the Y− chromosome), and the introduction of an Sry translocation onto an autosome (66, 67). FCG rodents give birth to four types of offspring: those with XX and XY chromosomes carrying the Sry gene and subsequently developing testes, and those with XX and XY chromosomes lacking Sry resulting in ovaries (68). Through the utilization of the FCG model, scientists have had the opportunity to investigate how gonadal hormones and sex chromosomes influence behaviors like ethanol intake, preference, and relapse-like behavior (69). Additionally, the model reveals insights into sex differences in brain structure and function (70, 71), cardiovascular responses (72), and sex-specific gene expression (73–75) thus highlighting the importance of sex chromosomes in understanding health and disease disparities. Additionally, RSPO1 has been identified within the brain, at higher levels within the male hypothalamus (76), and appears to be involved in food intake (77). Furthermore, research has indicated direct effects of Sry within the brain. Indeed, Sry protein has been found within the hypothalamus and cortical areas of adult men (78). Studies investigating the functional role of Sry within the brain have colocalized Sry within Tyrosine Hydroxylase (TH) producing cells, and antisense interference of Sry mRNA levels results in lowered TH levels (79). Whereas, increasing the levels of Sry results in increased dopamine levels. As males have higher TH levels in some brain regions, it is plausible that Sry directly contributes to sex differences within the brain. These data indicate that Sry not only influences gonadal development, but is also expressed within the brain to directly influence brain development.

Additionally, the *Gli2*+/−; *Gli3Δ*699/+ model offers a method for exploring the connection between brain and peripheral physiology in both males and females due to the importance GLI proteins have on the development of both peripheral and central tissues (80, 81). Specifically, Gli3 loss of function impairs Gonadotropin-releasing hormone (GnRH) cell migration into the rodent hypothalamus. In humans, this altered GnRH migration pattern can result in Kallmann syndrome (KS), a disorder resulting in anosmia and impaired puberty, as GnRH signaling is unable to properly signal. This phenomenon in rodents has been reported in an individual with KS that had a frameshift within GLI3 that resulted in a TGA stop codon. These data further elucidate that genes involved in urogenital development also impact brain development. Importantly, understanding this connection can be of a clinical benefit in addressing mental health concerns specific to VSCs populations, as the *Gli2*+/−; *Gli3Δ*699/+ model can help discerning whether the elevated levels of mental health distress in the VSC community (82) are of a genetic or environmental/societal origin.

### Connections to clinical practice

Research on sexual differentiation of the brain in animal models provides a critical foundation for translating basic neuroendocrine mechanisms into clinical understanding and care for individuals with VSCs. Rodent and nonhuman primate studies have revealed that exposure to androgens and estrogens during defined developmental windows permanently organizes neural circuits that govern reproductive behavior, stress reactivity, and socioemotional processing. These findings have clinical relevance for understanding the diversity of neurodevelopmental and psychological profiles observed among individuals with VSCs, many of whom report higher rates of anxiety, depression, and altered stress responsivity. By mapping how specific hormonal milieus shape neural architecture and function, animal models guide clinicians in identifying neuroendocrine pathways that may underlie these experiences and in tailoring therapeutic approaches accordingly.

Insights from rodent models, such as those manipulating perinatal testosterone or estradiol exposure, have informed the concept of organizational vs. activational effects of hormones, principles now applied clinically to understand how variations in prenatal or pubertal hormone exposure may influence later cognitive and emotional outcomes. Similarly, studies using the Four Core Genotype (FCG) mouse model have shown that sex chromosome complement can independently shape brain development, gene expression, and behavior, providing a mechanistic framework for interpreting clinical phenotypes that cannot be fully explained by hormonal differences alone. These data have refined genetic counseling and informed more precise categorization of neurodevelopmental risks associated with sex chromosome variations.

Translational value also extends to models that connect peripheral and central physiology, such as *Gli2/Gli3* mutant mice, which link urogenital and neurodevelopmental defects through disrupted GnRH neuronal migration. These findings have clinical parallels in conditions where reproductive and olfactory deficits coexist, and highlight the intertwined nature of neuroendocrine development across organ systems. Understanding these shared developmental origins helps clinicians anticipate comorbidities, improving diagnostic accuracy and interdisciplinary management.

Moreover, nonhuman primate models, which more closely mirror human patterns of brain sexual differentiation and pubertal timing, are particularly valuable for bridging preclinical findings to human application. Their use has enhanced understanding of how timing of hormonal exposure, whether during fetal, perinatal, or pubertal periods, affects cognitive flexibility, social interaction, and affective regulation. By continuing to refine translational bridges from animal models to clinical frameworks, researchers and clinicians can better support both the physiological and psychological well-being of individuals with VSCs.

### Limitations and future directions

While rodent models have been invaluable for delineating the genetic and hormonal mechanisms underlying brain sexual differentiation, they present notable limitations for translational research. In rodents, sexual differentiation of the brain occurs primarily postnatally through aromatization of testosterone into estradiol, whereas in primates and humans it takes place prenatally and largely under direct androgenic influence, with distinct placental aromatase activity and sex hormone-binding globulin profiles (83, 84). Moreover, the Four Core Genotype (FCG) mouse model, though powerful for dissociating chromosomal from gonadal effects, is limited by autosomal Sry (85) overexpression and strain-specific background effects (68). Similarly, Gli2/Gli3 mutants, which model disrupted GnRH neuronal migration analogous to Kallmann syndrome, often exhibit early lethality or anosmia that prevent behavioral assessment into adulthood.

To overcome these limitations, a comparative, multi-species framework is essential. Nonhuman primates (NHPs) provide the closest translational bridge to humans due to shared neuroanatomical structures, extended postnatal brain maturation, and parallel endocrine trajectories. Studies in rhesus macaques (*Macaca mulatta*) demonstrate that mid-gestational androgen exposure organizes neural circuits underlying sociosexual behaviors, directly informing human models of androgen-related neurodevelopment (86). Similarly, the common marmoset (*Callithrix jacchus*), with its cooperative breeding, biparental care, and rich vocal repertoire, offers a powerful small-primate model for investigating how pubertal steroids influence social communication, affective regulation, and cognitive flexibility (87).

Beyond primates, other animal taxa provide complementary mechanistic insights that extend the scope of VSC-related neuroendocrine research. Songbirds, for instance, exhibit striking sexual dimorphisms in vocal control nuclei and serve as valuable models for understanding hormone-dependent neuroplasticity, social communication, and learning (58). Moles and voles, with naturally occurring sex-reversed or socially monogamous phenotypes, enable examination of how alternative steroid environments and social structures shape neural circuits involved in parental and affiliative behaviors (88). Reptiles and amphibians, whose sex differentiation often depends on environmental cues such as temperature or social context, present unique systems for studying epigenetic and developmental plasticity in sexual differentiation (89). Fish models, particularly zebrafish and medaka, offer high-throughput access to early neural differentiation and gene-editing approaches for dissecting steroid receptor and aromatase function (90). Each of these taxa contributes distinct comparative and mechanistic strengths, together forming a continuum of models that illuminate conserved and divergent pathways shaping brain sexual differentiation.

Future directions should therefore embrace integrative, cross-species approaches that link molecular, endocrine, and behavioral endpoints across phylogeny. Combining the genetic tractability of rodents and fish with the developmental fidelity of primates, and the naturally variable sex differentiation systems of birds, reptiles, and amphibians, will yield a more complete understanding of how genetic and hormonal mechanisms converge to shape brain organization and behavior. Such a comparative strategy will not only refine translational relevance for clinical care of individuals with VSCs but also expand our broader understanding of sexual diversity as a fundamental feature of vertebrate neurobiology.

## Discussion (final conclusion)

Congenital conditions such as VSCs are among the most frequently observed in newborns, and their incidence is increasing (7). In males, conditions like cryptorchidism (failure of the testes to descend), hypospadias (misplacement of the urethral opening), and incomplete fusion of the scrotal sac are among the most commonly reported congenital presentations (91). In females, birth malformations typically present as congenital uterine anomalies (such as vaginal duplication), posing a threat to childbirth. Understanding the relationship between genes involved in VSCs and mental health risk are indeed valuable. The utilization of model organisms like mice, rats, and birds are indispensable tools for understanding complex biological phenomena, like the development of VSCs as well as variations in psychiatric risk. The expansion of these models has revealed critical insights into the genetic determinants of gonadal development, hormonal regulation, and brain sexual differentiation. By leveraging genetic manipulations, hormonal interventions, and behavioral studies in model organisms, researchers can gain valuable insights into the underlying biology of VSCs. The insight should be used to establish better standards of care, and further breakdown barriers this community faces, and this improve the lives of those living with a VSC.
